# COVID-19 Is Distinct From SARS-CoV-2-Negative Community-Acquired Pneumonia

**DOI:** 10.3389/fcimb.2020.00322

**Published:** 2020-06-16

**Authors:** Yutian Zhou, Shujin Guo, Ye He, Qiunan Zuo, Danju Liu, Meng Xiao, Jinxiu Fan, Xiaohui Li

**Affiliations:** ^1^The Geriatric Respiratory Department of Sichuan Provincial People's Hospital, University of Electronic Science and Technology of China, Chengdu, China; ^2^The Respiratory Department of Wuhan Red Cross Hospital, Tongji Medical College of Huazhong University of Science and Technology, Wuhan, China

**Keywords:** COVID-19, clinical features, SARS-CoV-2, CAP, severity

## Abstract

**Background:** Corona virus disease (COVID-19) is an infectious respiratory disease that has spread rapidly across the world. Many studies have already evaluated the clinical features of COVID-19, but how it compares with severe acute respiratory syndrome coronavirus 2 (SARS-CoV-2)-negative community-acquired pneumonia (SN-CAP) is still unclear. Moreover, COVID-19 mortality is correlated with disease severity, but indicators for severity grading have not been specified. We aimed to analyze the clinical characteristics of COVID-19 in comparison with SN-CAP and find indicators for disease severity in COVID-19.

**Methods:** Patients diagnosed with COVID-19 and SN-CAP were enrolled. Clinical, radiological, and laboratory data were analyzed.

**Results:** The numbers of COVID-19 and SN-CAP patients enrolled were 304 and 138, respectively. The age of the patients was not significantly different between the groups. Compared with SN-CAP, COVID-19 patients had more symptoms of fever and dyspnea; and showed significant difference in blood count results. Computed tomography (CT) imaging of COVID-19 patients showed patchy ground-glass opacities that correlated with disease severity, whereas the CT imaging of SN-CAP patients showed patchy high-density shadows. COVID-19 patients were classified into moderate, severe, and critically severe groups. The severe and critically severe groups had elevated levels of white blood cells (WBC), neutrophils, platelets, C-reaction protein (CRP), lymphocyte ratio (NLR), platelet to lymphocyte ratio (PLR), troponin-I, creatinine, and blood urea nitrogen (BUN). However, they had decreased levels of lymphocytes, lymphocyte ratio, and albumin. Compared with the younger patients, the older COVID-19 individuals had more chronic diseases and significantly elevated levels of WBC, neutrophil, and CRP levels.

**Conclusion:** SN-CAP showed more inflammatory reaction than COVID-19. Old people with chronic diseases are more susceptible to COVID-19 and have a high likelihood of developing severe and critically severe infection. Levels of WBC, lymphocytes, neutrophils, CRP, NLR, PLR, troponin-I, creatinine, and BUN are important indicators for severity grading in COVID-19.

## Introduction

In December 2019, pneumonia cases associated with a novel coronavirus were registered in Wuhan City, Hubei Province of China (World Health Organization, 2020b; Zhu et al., 2020). On February 11, 2020, the International Committee on Taxonomy of Viruses (ICTV) named the novel virus severe acute respiratory syndrome coronavirus 2 (SARS-CoV-2), while the World Health Organization (WHO) declared “coronavirus disease” (COVID-19) as the official name of the disease caused by the virus. This followed an earlier declaration by the WHO on January 31, 2020, that had designated coronavirus disease a public health emergency of international concern. The Chinese government-sponsored research activities to evaluate the genetic and clinical features of the infection provided comprehensive guidelines on disease epidemiology, etiology, diagnosis, treatment, nursing, and infection control for the hospital and community settings. However, the number of infections continued to increase exponentially, causing widespread fear and panic in the nation (Huang et al., 2020; Li et al., 2020; She et al., 2020). The disease started to spread from China to other nations, prompting the WHO, on February 28, 2020, to raise the alarm of COVID-19 being a very high-risk disease (Huang et al., 2020). As of April 29, 2020, the world had confirmed at least 3,250,000 COVID-19 cases and 220,000 deaths, with both morbidity and mortality still rising.

The main pathogens for community-acquired pneumonia (CAP) are *Streptococcus pneumoniae, Hemophilus influenzae, Mycoplasma* pneumoniae, Chlamydophila legionella, virus (rhinovirus, adenovirus, coronavirus), and fungus (Metlay and Waterer, 2020). Unlike the common coronavirus, SARS-CoV-2 is highly contagious. It is important to distinguish COVID-19 from other types of CAP. Few studies have compared the clinical features of COVID-19 and other pneumonia. One study enrolled 19 COVID-19 and 15 other pneumonia patients, but the results may lack reliability due to the small sample analyzed (Zhao et al., 2020); another study analyzed the respective CT imaging features (Shi et al., 2020). The differences between COVID-19 and SN-CAP are still unclear. Therefore, distinction analysis is urgently needed for clinicians.

According to the Chinese Center for Disease Control and Prevention (China CDC) (She et al., 2020), the mortality from COVID-19 in China stands at 2.3%. Studies have shown that most patients have good prognosis, apart from older adults, who have fatal or near-fatal outcomes (Jin et al., 2020). Lymphopenia is an important symptom of COVID-19 (Huang et al., 2020); however, the indicators for disease severity grading are unclear. In this study, we aimed to analyze the clinical features of patients diagnosed with COVID-19 in Wuhan Red Cross Hospital and compare the clinical characteristics of COVID-19 to those of SN-CAP. Furthermore, we analyzed the clinical characteristics based on patient age, split into young age (18–44 years), middle age (45–59 years), and old age (≥60 years), and identified indicators for severity grading in moderate, severe, and critically severe patients.

## Materials and Methods

### Patients

In this retrospective study, cases diagnosed with COVID-19 according to WHO guidance (World Health Organization, 2020a) in Wuhan Red Cross Hospital from February 1, 2020, to March 15, 2020, and cases diagnosed with SN-CAP in Sichuan Provincial People's Hospital from February 1, 2020, to April 15, 2020, were enrolled. SN-CAP patients were negative for SARS-CoV-2, influenza A (H1N1), and influenza B virus. This retrospective study was approved by the ethics committee of Sichuan Provincial People's Hospital.

### Diagnostic Criteria and Disease Severity Grading Criteria

According to the WHO guidance, the patients were divided into young age (18–44 years), middle age (45–19 years), and old age (>60 years) groups.

According to the fifth edition of the China Guidelines for the Diagnosis and Treatment Plan of COVID-19 Infection by the National Health Commission (Trial Version 5) (Lin and Li, 2020), the cases were classified into four types: (1) mild: with slight clinical symptoms but no imaging presentations of pneumonia; (2) moderate: with fever, respiratory symptoms, and imaging presentations of pneumonia; (3) severe: with any of the following: respiratory distress with RR>30 time/min, oxygen saturation at rest <93%, or PaO2/FiO2 <300 mmHg(I mmHg = 0.133 kPa); (4) critically severe: with any of the following: respiratory failure needing mechanical ventilation, shock, or combination with other organ failure needing ICU intensive care. The mild type was not admitted to hospital, so we enrolled moderate, severe, and critically severe cases.

SN-CAP cases were diagnosed according to the American Thoracic Society/Infectious Diseases Society of America 2019 guideline (Metlay and Waterer, 2020) and divided into moderate and severe groups.

### Data Collection and Statistical Analysis

Clinical symptoms, radiological features, and laboratory examination data were collected from patients' electronic medical records. The data were reviewed by three physicians.

Data analyses were performed by SPSS software (Version 23.0, IBM, China). Continuous variables were measured as mean (standard deviation, SD). Categorical data were measured as number (%) and tested with Chi-Square test. One-way analysis of variance (ANOVA) was used to evaluate comparisons between the groups. *P* < 0.05 was considered statistically significant.

## Result

### Clinical Features in COVID-19 and SN-CAP

We enrolled 304 patients infected with SARS-CoV-2 with a mean age of 61.5 years (SD, 13.3 years). The gender composition was 166 females and 138 males. Besides, we included 138 SN-CAP patients who were negative for SARS-CoV-2; their gender ratio was 56 females to 82 males. The two study groups had no statistically significant difference in age distribution. At admission, both COVID-19 and SN-CAP patients presented with fever, cough, dyspnea, fatigue, chest distress, expectoration, sore throat, and diarrhea. However, COVID-19 patients had a higher rate of fever and dyspnea and a lower rate of expectoration than SN-CAP patients. Notably, 39 COVID-19 patients and 6 SN-CAP patients were asymptomatic at admission. According to the guidance, 140 COVID-19 patients were classified into the moderate group, 123 into the severe group, and 41 into the critically severe group; 97 SN-CAP patients were graded to the moderate group, and 41 to the severe group. We observed hypertension, coronary arteriosclerosis disease (CAD), diabetes, chronic obstructive pulmonary disease (COPD), renal failure, and malignant tumor as the most common complications in both groups. However, COVID-19 patients had a lower rate of COPD and malignancy than SN-CAP patients (Table 1).

**Table 1:** Clinical characteristics of COVID-19 and SN-CAP.

	**COVID-19** ***n* = 304**	**SN-CAP** ***n* = 138**	***P*-value**
Age, mean (SD), y	61.5(13.3%)	61.6(16.1)	0.921
Female	166 (54.61%)	56(40.58%)	<0.01
Male	138 (45.39%)	82(59.42%)	
**Signs and symptoms at admission, patient no**			
Fever	172 (56.58%)	42 (30.43)	<0.01
Cough	134 (44.08%)	74 (53.62)	0.06
Dyspnea	29 (9.54%)	3 (2.17%)	<0.01
Fatigue	32 (10.53)	5 (3.62%)	0.02
Chest distress	24 (7.89%)	3 (2.17%)	0.02
Expectoration	10 (3.29%)	53 (38.41%)	<0.01
Sore throat	5 (1.64%)	5 (3.62%)	0.2
Diarrhea	5 (1.64%)	1 (0.72%)	0.4
Asymptomatic	39 (12.83%)	6 (4.35%)	<0.01
**Chronic medical illness, patient no**			
Hypertension	83 (27.3%)	34 (24.64%)	0.56
CAD	21 (6.91%)	8 (5.8%)	0.66
Diabetes	40 (13.16%)	25 (18.12%)	0.17
COPD	7 (2.3%)	27 (19.57%)	<0.01
Renal failure	27 (8.88%)	18 (13.04%)	0.18
Malignancy	3 (0.99%)	15 (10.87%)	<0.01
**Laboratory result abnormalities, patient no**			
WBC count, <3.7 × 109/L	42 (13.82%)	4 (2.9%)	<0.01
Lymphocyte count, <0.8 × 109/L	97 (41.91%)	68 (49.28%)	<0.01
Lymphocyte ratio <20%	134 (44.08%)	93 (67.39%)	<0.01
Neutrophil count, x109/L	51 (16.78%)	37 (26.81%)	0.01
Platelet <85 × 109/L	15 (4.93%)	7 (5.07%)	0.95
CRP >10 mg/L	127 (41.78%)	98 (71.01%)	<0.01
Albumin <35 g/L	139 (45.72%)	95 (68.84%)	<0.01
ALT/AST abnormal	99 (32.57%)	42 (30.43%)	0.66
Creatinine >73 μmol/L	60 (19.74%)	28 (20.29%)	0.89
BUN, >8 mmol/L	87 (28.62%)	30 (21.74%)	0.13
LDH >250 U/L	42 (13.82%)	60 (43/48%)	<0.01
Creatine kinase >195 U/L	21 (6.91%)	6 (4.35%)	0.3
Troponin-I >0.4 ug/L	49 (16.12%)	25 (18.12%)	0.6
Patients tested for procalcitonin, no.	31	117	
Procalcitonin >0.05 ng/mL	13 (41.94%)	55 (47.01%)	0.61

*P-value indicates differences between COVID-19 and SN-CAP, P < 0.05 was considered statistically significant*.

Comparison of the blood cell and biochemistry results of the COVID-19 and SN-CAP patients revealed significance differences in the WBC count, lymphocyte ratio, and neutrophil count. In the subgroup analysis, we compared the COVID-19 and SN-CAP patients according to disease severity. In the moderate group comparison, the SN-CAP showed significantly elevated WBC count, neutrophil count, CRP, neutrophil to lymphocyte ratio (NLR), and platelet to lymphocyte ratio (PLR) but a decreased lymphocyte ratio. The severe and critically severe groups of COVID-19 were amalgamated into one group when compared with the SN-CAP severe group, and the results showed significance differences in the WBC count and neutrophil count (Table 2). The blood cell counts of the young age and middle age groups did not differ across the two study populations. However, we observed significant differences in the WBC count, lymphocyte ratio, and neutrophil count in the old age group (Supplementary Table 1).

Table 2Laboratory results of COVID-19 and SN-CAP.

**Table:** 

**mean (SD)**	**COVID-19** ***n* = 304**	**SN-CAP** ***n* = 138**	***P*-value**
White blood cell count, x109/L	6.47 (3.05)	8.13 (4.12)	<0.01
Lymphocyte count, x109/L	1.18 (0.72)	1.21 (0.69)	0.7
Lymphocyte ratio, %	20 (11)	17 (10)	<0.01
Neutrophil count, x109/L	4.82 (3.04)	6.25 (4.09)	<0.01
Platelet count, x109/L	204 (83)	199 (83)	0.52
C-Reactive protein, mg/L	45.6 (64.4)	40.5 (50.6)	0.44
NLR, %	6.75 (11.25)	8.55 (12.76)	0.12
PLR, %	225 (162)	227 (176)	0.82
Albumin, g/L	34.04 (4.68)	36.31 (6.61)	0.1
Troponin-I, ug/L	0.18 (0.63)	0.21 (0.42)	0.73
Creatinine, μmol/L	174 (320)	149 (297)	0.43
BUN, 8 mmol/L	7.36 (6.16)	6.73 (5.6)	0.31

**Table:** 

**Disease severity**	**Moderate**		P^*^	**Severe (severe** **+** **critically severe)**		P^**^
	**COVID-19** ***n*** **=** **140**	**SN-CAP** ***n*** **=** **97**		**COVID-19*****n*** **=** **164**	**SN-CAP** ***n*** **=** **41**	
White blood cell count, x109/L	5.72 (2.03)	7.67 (3.1)	<0.01	7.11 (3.58)	9.2 (5.85)	<0.01
Lymphocyte count, x109/L	1.44 (0.6)	1.27 (0.65)	0.04	0.96 (0.75)	1.06 (0.75)	0.43
Lymphocyte ratio, %	26.59 (10.17)	18 (0.95)	<0.01	15.39 (9.82)	13.85 (10.3)	0.37
Neutrophil count, x109/L	3.77 (1.82)	5.62 (2.74)	<0.01	5.73 (3.55)	7.71 (5.99)	<0.01
Platelet count, x109/L	223 (76)	187 (81)	0.01	188 (86)	203 (90)	0.33
C-Reactive protein, mg/L	16 (30)	34.88 (43.25)	<0.01	67.75 (73.93)	53.4 (63.1)	0.26
NLR, %	3.1 (2.41)	6.42 (8.14)	<0.01	11.66 (27.66)	13.6 (19)	0.67
PLR, %	181 (97)	193 (120)	0.42	262 (196)	309 (248)	0.2

P-value indicates differences between COVID-19 and SN-CAP; P^*^ indicates differences of moderate pneumonia between COVID-19 and SN-CAP; P^**^ indicates differences between COVID-19 severe + critically severe group and SN-CAP severe group.

*P < 0.05 was considered statistically significant*.

**Figure 1:**
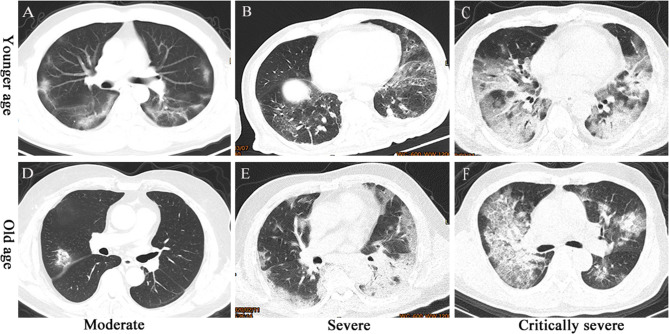
CT imaging of COVID-19 patients. **(A)** Moderate-severity pneumonia in a 36-year-old man presenting as ground-glass opacity under the pleura in both the lungs. **(B)** Severe pneumonia in a 50-year-old man presenting as ground-glass opacity and large exudative shadows in both the lungs. **(C)** Critically severe pneumonia in a 58-year-old man presenting as large patches of exudative shadows in both lungs. **(D)** Moderate-severity pneumonia in a 63-year-old woman presenting as ground-glass opacity in the right upper lobe. **(E)** Severe pneumonia in a 78-year-old woman presenting as large exudative shadows in both lungs. **(F)** Critically severe pneumonia in a 69-year-old man presenting as large patches of ground-glass opacity and exudative shadows in both lungs.

**Figure 2:**
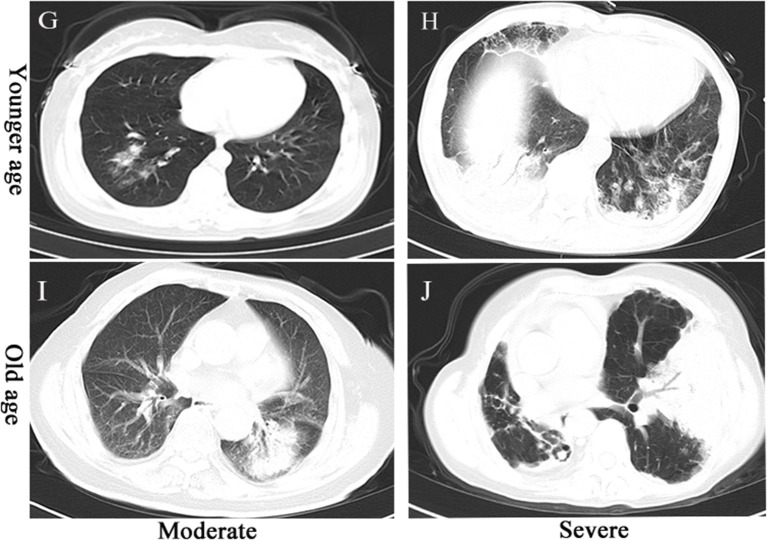
CT imaging of SN-CAP patients. **(G)** Moderate-severity pneumonia in a 49-year-old woman presenting as patchy exudative shadows in the right upper lobe. **(H)** Severe pneumonia in a 50-year-old man presenting as large exudative shadows in both lungs. **(I)** Moderate-severity pneumonia in a 90-year-old man presenting as exudative shadows in the left lower lobe. **(J)** Severe pneumonia in an 80-year-old woman presenting as large exudative shadows in both lungs.

Computed tomography (CT) imaging of COVID-19 patients showed mainly patchy ground-glass opacities under the pleura. These patchy shadows did not differ across the various age ranges. The severe and critically severe groups showed larger patchy and exudative shadows than the moderate patient group (Figure 1). Among SN-CAP patients, we observed patchy exudation in the lung lobes, and these features were not significantly different across the age ranges. The severe groups showed larger exudative shadows than the moderate group (Figure 2).

### Subgroup Comparisons of Clinical Features in COVID-19

The age range composition of COVID-19 was 43 young age, 88 middle age, and 173 old age patients. The young and middle age patients were amalgamated into one group when compared with the old age patients. Symptoms at admission did not differ across the various age ranges; however, the old age individuals had more chronic diseases (Table 3).

**Table 3:** Clinical characteristics of COVID-19 in different age ranges.

**Age range**	**Non-old (young and middle age), patient no**.					**Old age, patient no**.			
	**Total** ***n* = 137**	**Moderate** ***n* = 77**	**Severe** ***n* = 41**	**Critically severe** ***n* = 19**	***P*-Valve^**d**^**	**Total** ***n* = 167**	**Moderate** ***n* = 63**	**Severe** ***n* = 73**	**Critically severe** ***n* = 31**
Female	72 (52.55%)	45 (58.44%)	19 (46.34%)	8 (42.11%)	0.52	94 (56.29%)	40 (63.49%)	42 (57.53%)	12 (38.71%)
Male	65 (47.44%)	32 (41.56%)	22 (53.66%)	11 (57.89%)		73 (43.71%)	23 (36.51%)	31 (42.47%)	19 (61.29%)
**SIGNS AND SYMPTOMS AT ADMISSION**									
Fever	77 (56.20%)	43 (55.84%)	28 (68.29%)	6 (31.58%)	0.91	95 (56.89%)	32 (50.79%)	52 (71.23%)	11 (35.48%)
Cough	58 (42.34%)	36 (46.75%)	17 (41.46%)	5 (26.31%)	0.58	76 (45.51%)	27 (42.86%)	39 (53.42%)	10 (32.26%)
Dyspnea	9 (6.57%)	6 (7.79%)	2 (4.88%)	1 (5.26%)	0.11	20 (11.98%)	7 (11.11%)	8 (10.96%)	5 (16.13%)
Fatigue	19 (13.87%)	11 (14.29%)	6 (14.63%)	2 (10.53%)	0.09	13 (7.78%)	4 (6.35%)	8 (10.96%)	1 (3.23%)
Chest distress	10 (7.30%)	7 (9.09%)	1 (2.44%)	2 (10.53%)	0.75	14 (8.38%)	10 (15.87%)	2 (2.74%)	2 (6.45%)
Expectoration	6 (4.38%)	2 (2.60%)	3 (7.32%)	1 (5.26%)	0.33	4 (2.40%)	1 (1.59%)	2 (2.74%)	1 (3.23%)
Sore throat	3 (2.19%)	1 (1.30%)	2 (4.88%)	0	0.5	2 (1.20%)	1 (1.59%)	1 (1.37%)	0
Diarrhea	2 (1.46%)	1 (1.30%)	1 (2.44%)	0	0.82	3 (1.80%)	0	3 (4.11%)	0
Asymptomatic	19 (13.87%)	13 (16.88%)	6 (14.63%)	0	0.62	20 (11.98%)	10 (15.87%)	10 (13.70%)	0
**CHRONIC MEDICAL ILLNESS**									
Hypertension	18 (13.14%)	5 (6.49%)	11 (6.83%)	2 (10.53%)	<0.01	65 (38.92%)	21 (33.33%)	34 (46.58%)	10 (32.26%)
CAD	4 (2.92%)	1 (1.30%)	3 (7.32%)	0	0.01	17 (10.18%)	5 (7.94%)	10 (13.70%)	2 (6.45%)
Diabetes	7 (5.11%)	3 (3.90%)	2 (4.88%)	2 (10.53%)	<0.01	33 (19.76%)	11 (17.46%)	16 (21.92%)	6 (19.35%)
COPD	1 (0.73%)	1 (1.30%)	0	0	0.1	6 (3.59%)	1 (1.59%)	4 (5.48%)	1 (3.23%)
Renal failure	12 (8.76%)	1 (1.30%)	9 (21.95%)	2 (10.53%)	0.95	15 (8.98%)	1 (1.59%)	11 (15.07%)	3 (9.68%)
Malignancy	2 (1.46%)	1 (1.30%)	1 (2.44%)	0	0.45	1 (0.60%)	0	1 (1.37%)	0

*P-value ^d^indicates differences between old age and non-old age COVID-19 patients, P < 0.05 was considered statistically significant*.

The COVID-19 patients registered declines in lymphocyte count, lymphocyte ratio, and platelet count. (Table 4). We did not find any significant difference in blood cell count between young age, middle age, and old age patients (Supplementary Table 2), but a significant disparity was evident between the moderate, severe, and critically severe groups. Compared with the moderate group, the severe and critically severe groups showed significant rises in WBC count, neutrophil count, NLR, PLR, CRP, lactate dehydrogenase (LDH), troponin-I, and creatinine and significant decreases in lymphocytes, lymphocyte ratio, platelets, and albumin (Table 4).

**Table 4:** Laboratory results of COVID-19.

	**Moderate *n* = 140**	**Severe *n* = 123**	**Critically severe** ***nn* = 41**	***P*-value^**a**^**	***P*-value^**b**^**	***P*-value^**c**^**
Age, mean (SD), y	55.9 (14.4)	63.8 (13.9)	65.2 (12.7)	0.06	0.05	0.06
Female	85	65	16	0.25	0.01	0.2
Male	55	58	25			
WBC, x109/L	5.72 (2.03)	6.47 (2.8)	8.97 (4.89)	0.03	<0.01	<0.01
Lymphocyte, x109/L	1.44 (0.6)	1.05 (0.8)	0.68 (0.44)	<0.01	<0.01	<0.01
Lymphocyte ratio, %	26.59 (10.17)	17.23 (8.64)	10.55 (11.44)	<0.01	<0.01	<0.01
Neutrophil, x109/L	3.77 (1.82)	5.02 (2.7)	7.86 (4.82)	<0.01	<0.01	<0.01
Platelet, x109/L	223 (76)	193 (85)	173 (91)	<0.01	<0.01	0.16
CRP, mg/L	16 (30)	46.2 (56.5)	114.8 (87.94)	<0.01	<0.01	<0.01
NLR, %	3.1 (2.41)	6.92 (6.3)	19.47 (24.35)	<0.01	<0.01	<0.01
PLR, %	181 (97)	234 (169)	346 (243)	<0.01	<0.01	<0.01
Albumin, g/L	35.73 (3.96)	33.68 (4.81)	29.48 (3.14)	0.002	<0.001	0.553
Troponin-I, ug/L	0.058 (0.17)	0.07 (0.11)	0.77 (1.35)	0.921	<0.001	0.475
Creatinine, umol/L	51.31 (36.62)	226.39 (384.2)	249.2 (348)	<0.001	<0.001	0.317
BUN, mmol/L	5.48 (2.9)	8.17 (7.12)	11.32 (8.29)	<0.001	<0.001	0.834

*P-value^a^ indicates differences between moderate and severe group; P-value^b^ indicates differences between moderate and critically severe group; P-value^c^ indicates differences between severe and critically severe group; P < 0.05 was considered statistically significant*.

In the young age group, we did not observe any significant difference in blood cell data across the moderate, severe, and critically severe groups. However, the blood cell data (WBC, lymphocyte, lymphocyte ratio, neutrophil, NLR, PLR, CRP) for the middle and old age groups differed significantly across the three categories of disease severity (Table 5).

**Table 5:** Blood cell analysis of COVID-19.

	**Moderate**	**Severe**	**Critically severe**	***P*-value^**a**^**	***P*-value^**b**^**	***P*-value^**c**^**
**YOUNG AGE: 18–44**						
Patient no. (43)	30	10	3			
WBC, x109/L	6.19 (2.2)	6.49 (2.79)	6.05 (2.67)	0.4	0.91	0.57
Lymphocyte, x109/L	1.55 (0.5)	1.05 (0.8)	0.79 (0.44)	0.22	0.03	0.18
Lymphocyte ratio, %	26.42 (7.8)	16.98 (8.69)	16.5 (14.82)	0.03	0.06	0.63
Neutrophil, x109/L	4.11 (1.76)	5.02 (2.69)	4.79 (2.62)	0.16	0.53	0.84
Platelet, x109/L	238 (74)	194 (84)	120 (36)	<0.05	0.01	0.22
CRP, mg/L	12 (22.8)	67.77 (56.75)	64.84 (71.12)	0.27	0.1	0.08
NLR, %	2.81 (1.33)	9.08 (28.29)	9.51 (9.8)	0.04	<0.01	0.03
PLR, %	165 (58)	234 (170)	182 (82)	0.89	0.74	0.82
**MIDDLE AGE: 45–59**						
Patient no. (88)	44	33	11			
WBC, x109/L	5.38 (2.3)	7.19 (3.17)	8.27 (4.96)	0.01	<0.01	0.31
Lymphocyte, x109/L	1.31 (0.55)	0.99 (0.37)	0.67 (0.54)	<0.01	<0.01	0.07
Lymphocyte ratio, %	26.16 (11.62)	15.05 (7.64)	10.44 (10.24)	<0.01	<0.01	0.19
Neutrophil, x109/L	3.6 (2.2)	5.64 (3.09)	7.37 (4.63)	<0.01	<0.01	0.09
Platelet, x109/L	225 (86)	214 (103)	143 (53)	0.6	<0.01	0.03
CRP, mg/L	19.22 (40.53)	67.58 (82.27)	105 (96)	0.01	<0.01	0.14
NLR, %	3.34 (2.82)	6.97 (6.1)	14.98 (8.74)	<0.01	<0.01	<0.01
PLR, %	198 (110)	245 (139)	300 (185)	0.13	0.02	0.23
**OLD AGE: ≥60**						
Patient no. (173)	66	80	27			
WBC, x109/L	5.74 (1.74)	6.32 (2.75)	9.57 (5.02)	0.4	<0.01	<0.01
Lymphocyte, x109/L	1.47 (0.67)	1.09 (1.24)	0.67 (0.41)	<0.01	<0.01	0.03
Lymphocyte ratio, %	11.24 (14.44)	16.73 (8.28)	9,93 (11.81)	<0.01	<0.01	<0.01
Neutrophil, x109/L	3.72 (1.58)	4.97 (2.65)	8.4 (5.05)	0.03	<0.01	<0.01
Platelet, x109/L	215 (71)	190 (81)	191 (102)	0.03	0.19	0.81
CRP, mg/L	16.3 (26.07)	44.69 (42.17)	125 (87)	<0.01	<0.01	<0.01
NLR, %	3.25 (2.6)	20.81 (34.75)	22.41 (29.1)	0.1	<0.01	0.04
PLR, %	178 (101)	245 (187)	383 (267)	0.04	<0.01	<0.01

*P-value^a^ indicates differences between moderate and severe group; P-value^b^ indicates differences between moderate and critically severe group; P-value^c^ indicates differences between severe and critically severe group; P < 0.05 was considered statistically significant*.

Regarding the biochemistry data, the COVID-19 patients had a rise in C-reactive protein (CRP) levels. The young, middle, and old age COVID-19 patients had no significant difference in CRP levels. However, in terms of disease severity, the severe and critically severe patient groups had higher CRP levels than the moderate group. When disease severity was stratified according to age, we did not find a significant difference in CRP levels in the young patients. This relationship changed in the middle and old age groups, in which CRP levels differed significantly.

## Discussion

COVID-19 is a highly contagious disease that poses a serious threat to public health across the globe (Feng et al., 2020; Wang D. et al., 2020; Wu and McGoogan, 2020). Despite major investment, there is still a shortage of medical staff and resources. In order to optimize medical resources and ensure maximum patient care, it is essential to recognize the disease as distinct from CAP and identify the severe patients. Older adults had a higher severity of COVID-19 due to the low immunity status associated with the aging process (Applegate and Ouslander, 2020). Immunity is an essential factor for disease development and severity. These facts informed the stratification of the patients into young age, middle age, and old age groups for further comparison. SN-CAP had more symptoms of expectoration and higher WBC and neutrophil counts than COVID-19 due to increased infiltration of inflammation cells, injury of alveolar walls, and high inflammatory exudation in the alveoli. Pathogens of CAP mainly include bacteria, mycoplasma, virus, and fungus; however, bacterial infection accounts for more than half (Metlay and Waterer, 2020). Elevated WBC count, neutrophil count, and CRP level are the common inflammatory indicators in bacterial infection. According to disease severity, both the moderate and severe groups showed significant increases in many inflammatory indicators in SN-CAP. Patients of the two groups had no significant difference in chronic disease status, except COPD, and malignancy. This may be caused by the case enrollment: the SN-CAP patients were mainly enrolled from the respiratory department in Sichuan Provincial People's Hospital, and COPD and pulmonary tumor are important respiratory diseases in the department. Furthermore, the morbidity of COPD is high in Sichuan province, and the incidence of SN-CAP in these patients is higher in winter and spring.

CT imaging of COVID-19 patients showed patchy ground-glass opacities under pleura, and these findings are consistent with the findings of previous studies (Wang D. et al., 2020; Xu Y. H. et al., 2020). The CT presentation in the severe and critically severe groups showed larger patchy and exudative shadows, which represent the pathological alterations of SARS-CoV-2 or/and bacteria (Wang D. et al., 2020). The CT imaging of SN-CAP showed patchy high-density shadows, which were caused by inflammatory exudation. CT imaging presentation is an important indicator for the disease severity of both COVID-19 and SN-CAP.

According to the China CDC guidelines, COVID-19 was divided into mild, moderate, severe, and critically severe. In this study, we included patients with signs of pneumonia in CT imaging, and since mild cases have no pneumonia presentation, they were excluded. The blood count results of 140 moderate, 123 severe, and 41 critically severe COVID-19 patients were analyzed. The age showed no significant difference in these groups. Consistent with previous research, COVID-19 patients mainly presented with fever, cough, fatigue, and dyspnea (Yang et al., 2020; Zhang et al., 2020). Older COVID-19 individuals had more chronic diseases and significantly elevated WBC, neutrophil, and CRP levels.

Compared to the moderate group, the severe and critically severe groups had increased levels of WBC, neutrophil, platelets, CRP, NLR, PLR, BUN, LDH, troponin-I, and creatinine and decreased lymphocyte ratios and levels of lymphocytes and albumin. These abnormal findings for blood cells and biochemistry suggest that the virus infection may induce liver, kidney, and myocardial injury in addition to the destruction of immune cells (Wang C. et al., 2020). The study results are consistent with autopsy findings of COVID-19 patients, which have shown lung, liver, and myocardium injury. On histological examination, the lung tissue was characterized by inflammatory infiltrates and dominated by lymphocytes, while liver biopsy revealed microvesicular steatosis due to the direct effect of the virus or drugs. A few monocytes infiltrated the myocardium, causing pathological changes (Xu Z. et al., 2020). Possibly, the virus mainly induces inflammation in the lungs, as do SARS and MERS (Ding et al., 2003; Ng et al., 2016). In the young age group, we did not observe any significant difference in blood cell metrics across the moderate, severe, and critically severe groups. However, the values of the blood cell metrics (WBC, lymphocyte, lymphocyte ratio, neutrophil, NLR, PLR, and CRP) for the middle and old age groups differed significantly across the three categories of disease severity. This may be caused by the co-infection of bacteria in the middle and old age groups. Viruses interfere with the immune system of patients and then induce secondary bacterial infection (Du Toit, 2020). It had been reported that bacterial infection is common in H1N1 infection (Milosevic et al., 2013). Because of the low immunity, the middle age and old age individuals are more susceptible to secondary bacterial infection, especially patients with chronic disease.

Previous studies showed that lymphopenia is a typical feature in COVID-19 patients and might be associated with disease severity (Chan et al., 2020). In this study, nearly 30–40% of patients had decreased levels of lymphocytes and 50% had a decline in the lymphocyte ratio. Notably, lymphopenia was more remarkable in the severe and critically severe groups. The study findings, therefore, corroborated previous research that has identified lymphopenia as an important indicator of COVID-19 severity. Despite the lymphopenia status in the severe and critically severe groups, the WBC levels were elevated, possibly due to secondary bacterial infections ((Chen et al., 2020). Destruction of the immune cells by SARS-CoV-2 virus makes the patients vulnerable to secondary bacterial infections. Additional indicators include the NLR and PLR, which are sensitive biomarkers for both natural and acquired immune responses (Polat et al., 2014; Kartal and Kartal, 2017). They are considered indicators of infection and systemic inflammation (Korkmaz et al., 2015). In this study, both NLR and PLR were statistically different across the moderate, severe, and critically severe groups. The NLR and PLR values were closely correlated with the severity of the disease.

This study has several limitations. First, due to a shortage of medical staff and resources, sputum culture is impossible in Wuhan Red Cross Hospital, and thus there is no evidence with which to identify secondary bacterial infections. Second, COVID-19 patients were enrolled from Wuhan Red Cross Hospital, and SN-CAP patients were from Sichuan Provincial People's Hospital; multi-center studies with more patients are needed for further evaluation.

In summary, COVID-19 is a highly infectious disease that affects people across all age groups. Patients with COVID-19 had lower WBC and neutrophil levels than those with SN-CAP. The older adults with chronic diseases were more susceptible to severe and critically severe infections. CT imaging presentation, lymphopenia, CRP, NLR, and PLR are significant indicators for severity grading of COVID-19.

## Data Availability Statement

The original contributions presented in the study are included in the article files; further inquiries can be directed to the corresponding authors.

## Ethics Statement

The studies involving human participants were reviewed and approved by Ethics committee of Sichuan Provincial People's Hospital. Written informed consent for participation was not required for this study in accordance with the national legislation and the institutional requirements.

## Author Contributions

SG, YZ, and YH collected data. SG and QZ contributed to statistical analyses. DL, YZ, and JF analyzed CT images. SG and MX edited tables. SG and YH edited the manuscript. YZ and XL reviewed the manuscript. YZ and JF did their best to treat COVID-19 patients in Wuhan Red Cross Hospital. They fought for 2 months until the last patient was discharged from the hospital. All authors contributed to the article and approved the submitted version.

## Conflict of Interest

The authors declare that the research was conducted in the absence of any commercial or financial relationships that could be construed as a potential conflict of interest.
