# Supplementary material for: COVID-19 Is Distinct From SARS-CoV-2-Negative Community-Acquired Pneumonia
Source: Front Cell Infect Microbiol. 2020 Jun 16;10:322. doi: 10.3389/fcimb.2020.00322 (PMC7309449; doi:10.3389/fcimb.2020.00322)
Supplement: Supplementary file 1 [file Data_Sheet_1.docx]

| **Supplementary Table 1: Blood cell comparison according to age range in COVID-19 and SN-CAP** | | | |
| --- | --- | --- | --- |
|  | **COVID-19(304)** | **SN-CAP (138)** | **P-value** |
| **Young age: 18-44** | | | |
| Patient no. | 43 | 23 |  |
| White blood cell count, x109/L | 6.33(2.08) | 6.98(1.82) | 0.22 |
| Lymphocyte count, x109/L | 1.44(0.59) | 1.31(0.5) | 0.34 |
| Lymphocyte ratio, % | 24.06(9.1) | 20(9.18) | 0.09 |
| Neutrophil count, x109/L | 4.37(1.77) | 4.04(1.89) | 0.16 |
| Platelet count, x109/L | 216(81) | 208(80) | 0.68 |
| C-Reactive protein, mg/L | 19.57(34.1) | 21.22(22) | 0.9 |
| NLR, % | 3.82(3.48) | 5.04(4.47) | 0.22 |
| PLR, % | 167(80) | 183(97) | 0.48 |
| **Middle age: 45-59** | | | |
| Patient no. | 88 | 29 |  |
| White blood cell count, x109/L | 6.34(3.27) | 7.44(2.2) | 0.12 |
| Lymphocyte count, x109/L | 1.09(0.54) | 1.32(0.73) | 0.1 |
| Lymphocyte ratio, % | 20.13(11.83) | 18.71(9.88) | 0.5 |
| Neutrophil count, x109/L | 4.78(3.21) | 5.47(2.14) | 0.33 |
| Platelet count, x109/L | 208(95) | 193(69) | 0.37 |
| C-Reactive protein, mg/L | 51.77(75.67) | 49.63(60.43) | 0.86 |
| NLR, % | 6.09(6.36) | 5.48(4.48) | 0.6 |
| PLR, % | 226(137) | 176(101) | 0.06 |
| **Old age: ≥60** | | | |
| Patient no. | 173 | 86 |  |
| White blood cell count, x109/L | 6.53(3.17) | 8.66(4.95) | <0.01 |
| Lymphocyte count, x109/L | 1.15(0.82) | 1.14(0.71) | 0.92 |
| Lymphocyte ratio, % | 19.94(11.63) | 15.46(9.62) | <0.01 |
| Neutrophil count, x109/L | 4.92(3.21) | 6.83(4.84) | <0.01 |
| Platelet count, x109/L | 198(79) | 198(89) | 0.99 |
| C-Reactive protein, mg/L | 48.5(62.77) | 42.75(51.75) | 0.46 |
| NLR, % | 7.77(13.97) | 10.52(15.5) | 0.15 |
| PLR, % | 238(186) | 257(205) | 0.46 |

P-value indicates differences between COVID-19 and SN-CAP, P＜0.05 was considered statistically significant.

| **Supplementary Table 2: Blood cell analysis of COVID-19 according to age range** | | | | | | |
| --- | --- | --- | --- | --- | --- | --- |
| Age range, y | Young age  n = 43 | Middle age  n = 88 | Old age  n = 193 | P-value ^a^ | P-value ^b^ | P-value ^c^ |
| White blood cell count, x109/L | 6.33(2.08) | 6.34(3.27) | 6.53(3.17) | 0.88 | 0.7 | 0.78 |
| Lymphocyte count, x109/L | 1.44(0.59) | 1.09(0.54) | 1.15(0.82) | 0.01 | 0.02 | 0.67 |
| Lymphocyte ratio, % | 24.06(9.1) | 20.13(11.83) | 19.94(11.63) | 0.08 | 0.03 | 0.78 |
| Neutrophil count, x109/L | 4.37(1.77) | 4.78(3.21) | 4.92(3.21) | 0.41 | 0.29 | 0.81 |
| Platelet count, x109/L | 216(81) | 208(95) | 198(79) | 0.68 | 0.19 | 0.26 |
| CRP, mg/L | 19.57(34.1) | 51.77(75.67) | 48.5(62.77) | 0.02 | 0.02 | 0.7 |
| NLR, % | 3.82(3.48) | 6.09(6.36) | 7.77(13.97) | 0.26 | 0.04 | 0.27 |
| PLR, % | 167(80) | 226(137) | 238(186) | 0.04 | 0.01 | 0.66 |
| Albumin, g/L | 35.51(5,87) | 33.73(4.68) | 32.6(4.45) | 0.1 | <0.01 | 0.14 |
| Troponin-I, ug/L | 0.1(0.17) | 0.14(0.39) | 0.24(0.79) | 0.2 | 0.2 | 0.14 |
| Creatinine, umol/L | 144.84(358,67) | 206(336.73) | 169(295.62) | 0.3 | 0.65 | 0.38 |
| BUN, mmol/L | 4.86(4.96) | 7.66(5.99) | 7.69(6.25) | 0.01 | 0.01 | 0.97 |

P-value^a^ indicates differences between moderate and severe group; P-value^b^ indicates differences between moderate and critically severe group; P-value^c^ indicates differences between severe and critically severe group; P＜0.05 was considered statistically significant.
